# Statin-induced autoimmune myositis: a proposal of an “experience-based” diagnostic algorithm from the analysis of 69 patients

**DOI:** 10.1007/s11739-023-03278-9

**Published:** 2023-05-05

**Authors:** Carola Maria Gagliardo, Davide Noto, Antonina Giammanco, Silvia Maltese, Luca Vecchio, Giuseppe Lavatura, Valentina Cacciatore, Carlo Maria Barbagallo, Antonina Ganci, Emilio Nardi, Marcello Ciaccio, Rosalia Lo Presti, Angelo Baldassare Cefalù, Maurizio Averna

**Affiliations:** 1 Department of Health Promotion, Maternal and Child Health, Internal and Specialized Medicine of Excellence “G. D. Alessandro” (PROMISE), University of Palermo, Street: Via del Vespro 127, 90127 Palermo, Italy; 2 Complex Operating Unit of Nephrology and Dialysis, “San Giovanni Di Dio” Hospital of Agrigento, Agrigento, Italy; 3 Department of Biomedicine, Neuroscience and Advanced Diagnostics (BIND), University of Palermo, Palermo, Italy; 4 Department of Psychological, Pedagogical, Exercise and Training Sciences, University of Palermo, Palermo, Italy

**Keywords:** Statin-induced autoimmune myositis (SIAM), Anti-HMGCR antibody, SIAM case report, SIAM diagnostic algorithm, Muscle biopsy

## Abstract

Statin-induced autoimmune myositis (SIAM) represents a rare clinical entity that can be triggered by prolonged statin treatment. Its pathogenetic substrate consists of an autoimmune-mediated mechanism, evidenced by the detection of antibodies directed against the 3-hydroxy-3-methylglutaryl-coenzyme A reductase (anti-HMGCR Ab), the target enzyme of statin therapies. To facilitate the diagnosis of nuanced SIAM clinical cases, the present study proposes an “experience-based” diagnostic algorithm for SIAM. We have analyzed the clinical data of 69 patients diagnosed with SIAM. Sixty-seven patients have been collected from the 55 available and complete case records regarding SIAM in the literature; the other 2 patients represent our direct clinical experience and their case records have been detailed. From the analysis of the clinical features of 69 patients, we have constructed the diagnostic algorithm, which starts from the recognition of suggestive symptoms of SIAM. Further steps provide for CK values dosage, musculoskeletal MR, EMG/ENG of upper-lower limbs and, Anti-HMGCR Ab testing and, where possible, the muscle biopsy. A global evaluation of the collected clinical features may suggest a more severe disease in female patients. Atorvastatin proved to be the most used hypolipidemic therapy.

**Supplementary Information:**

The online version contains supplementary material available at 10.1007/s11739-023-03278-9.

## Introduction

Statin-induced autoimmune Myositis (SIAM) is a rare clinical entity that can occur as a side effect after prolonged statin treatment, with a prevalence of 1 in 100,000 inhabitants [1, 2]. A wide spectrum of musculoskeletal disorders associated with statin therapy is included in the definition of “Statin-induced myopathy spectrum” (SIMS). Clinical presentations may also range from asymptomatic elevation of creatine kinase (CK), sometimes associated with myalgia and exercise intolerance, to rarer forms characterized by proximal muscle weakness, marked serum CK elevation and histological evidence of myonecrosis. The necrotizing and autoimmune forms are commonly defined as “Statin-induced necrotizing autoimmune Myositis (SINAM) [3]. Symptoms and signs of SIMS also include fatigue, cramping, stiffness, tendon pain, tenosynovitis and rare cases of dysphagia. [4, 5]. Statin-induced myositis (SIM) recognizes two mechanisms: a more common cytotoxic mechanism, and a rarer autoimmune-mediated mechanism, associated with the presence of antibodies directed against the 3-hydroxy-3-methylglutaryl-coenzyme A reductase (Anti-HMGCR Ab). Normally, HMGCR is highly expressed in muscles and it represents the target enzyme on which statins act as competitive inhibitors, lowering the production of cholesterol [6]. The autoimmune-mediated mechanism represents the pathogenetic substrate of SIAM. However, the exact pathophysiological mechanism leading to SIAM remains unclear. It is hypothesized to include mechanisms affecting membrane excitability, mitochondrial function, ubiquinone depletion, calcium-metabolism, and apoptosis. [7]. The onset appears to be more common after the age of 50 years, with a slight preponderance for the female sex [2].

Side effects induced by statins usually are dealt with discontinuation of statin therapy. SIAM is instead later suspected when resolution or improvement of symptoms does not occur despite statin discontinuation. Moreover, SIAM-affected patients treated after a time delay (months or years) in some cases require a prompt immunosuppressive treatment [6].

In this article the authors summarize the most contemporary literature regarding SIAM and provide two illustrative case reports relevant to the topics discussed. To the best of authors’ knowledge, only a single SIAM diagnostic algorithm has been published so far [8]. Therefore, to facilitate the diagnosis of nuanced SIAM clinical cases, here we propose a modified diagnostic algorithm of SIAM. The development of the present algorithm takes into account data from a total of 69 patients, collected both from literature and from our personal experience.

## Methods

Two patients were admitted to the Internal Medicine Unit of the University Hospital of Palermo in the period 2021–2022. Patient’s clinical data were collected retrospectively; therapy and instrumental reports were searched in the medical records of patients’ hospitalizations. After hospital discharge, clinical data were collected through telephone interviews. Informed consents were obtained for both patients of the clinical cases in written and verbal forms. Declaration of Helsinki 1973. From April 2022 to May 2022 a literature search was carried out on PubMed, examining case records regarding SIAM. The search terms were: “Statin-induced autoimmune Myopathy Case report”, “Statin-induced autoimmune Myositis Case report”, “HMGCR myopathy Case report”, “HMGCR myositis Case report”. A total of 191 case reports concerning SIAM were identified by these terms, and a total of 121 articles remained after duplicate removal. Articles were then filtered by English language (17 articles removed) and full-text availability (15 articles removed. A total of 55 articles were selected according to the following inclusion criteria: (i) articles describing the patients' diagnostic *iter* and concerning only statin-induced forms of myositis with an autoimmune mechanism, (ii) incomplete clinical information of the patients, concerning statin-induced forms of myositis with cytotoxic mechanism and/or HMGCR-ab positive but paraneoplastic forms of myositis (Supplementary Fig. 1). As the work is not designed as a ‘systematic review’ of the literature, the methodological quality of the work was not assessed. Therefore, only 55 out of 191 articles were selected and used as a cohort of patients to derive overall data on the main clinical-therapeutic features of SIAM. Data regarding 67 patients were collected from the 55 publications and tabulated in a Microsoft Excel spreadsheet. Bibliographic sources are listed in Supplementary Table 1. A diagnostic algorithm was built according to the data obtained by the revision of the 69 cases.

## Results

### Cases description

#### Case 1

Case of a 67 years-old woman with a clinical history of chronic ischemic heart disease and hyperlipidemia, in treatment with beta-blockers, ACE-inhibitors, antiplatelet agents and atorvastatin 20 mg/day for 10 years. She referred her symptoms starting in April 2021 with progressive fatigue and muscle weakness in the lower limbs. In August of the same year, because of a marked serum elevation of CK [over 48 folds the Upper Limit of Normal (ULN) 15–250 U/L), atorvastatin was discontinued but due to the onset of a progressive and severe lower hyposthenia and dysphagia, she was admitted at our Division in September 2021. At the admission time, a proximal muscle atrophy of the lower limbs and reduced strength were noticed during the neurological examination. She was not capable of walking, lifting, or pushing her legs against minimal resistance from the examiner and she was unable to rise from a seated position. Skin inspection showed a mild rash limited to the upper back and trunk, without the involvement of the face. Blood chemistry showed, CK (31 folds the ULN, normal value (NV) 26–192 U/L), myoglobin (59 folds the ULN < 58 mcg/L U/L), aspartate and alanine aminotransferases GOT/GPT (6/4 folds the ULN, respectively, NV: 0–35 U/L) and LDH (2 folds the ULN, NV: 50–250 U/L). Inflammation and kidney function markers resulted within the normal range. Complete results of the tests are reported in Supplementary Table 2. The clinical presentation was suggestive of rhabdomyolysis and fluid therapy was immediately started. The successive exams were performed to make differential diagnoses among the various forms of rhabdomyolysis. Infectious and traumatic causes were excluded [9] and the acid alpha-glucosidase enzyme activity test (dried blood spot-DBS) was normal excluding Pompe Disease. The electromyography/electroneurography (EMG/ENG) detected a pattern of severe myopathy with signs of myotonia. The musculoskeletal magnetic resonance (MR) of the lower limbs reported a hyperintensity of the signal in the muscle gizzards bilaterally with imbibition of the subcutaneous soft tissues: these findings were compatible with an active inflammatory pattern and suggestive of myositis [10]. The total body computed tomography (CT) with contrast medium, the positron emission tomography scan (PET/CT), the pelvic echography and the tumor markers (Carcinoembryonary antigen- CEA, CA15-3, CA 19-9, alpha-fetoprotein, CA 125) within the normal range excluded the paraneoplastic etiology of the myositis [11]. The autoimmune origin was investigated through the research of specific myositis autoantibodies panel [12]: Anti-Nuclear Antibodies (ANA) resulted in mildly positive with 1:80 antibody dilution; autoantibodies anti-Mi2/alpha and beta nucleosome remodeling and deacetylase complex, anti-DNA, SRP (signal recognition particle), PL7 (threonyl), PL-12 (alanyl), EJ (glycyl), OJ (isoleucyl), Jo 1 (antihistidyl-tRNA synthetase), PM-Scl100 and PM-Scl75, Ro-52, NXP2, TIF 1g, SAE1 and MDA5 resulted negative. Only a weak positivity was noticed for anti-Ku antibodies. Anti-HMGCR Ab exceeded 3 folds of the ULN so the diagnosis of SIAM was made. The first line of therapy consisted of high-dose steroid therapy with intravenous prednisone 1 mg/kg body weight/day, followed after two weeks by one cycle of intravenous human immune globulin (IVIG) at the dosage of 400 mg/Kg. One month after the beginning of therapy, given the absence of clinical improvement, a weekly injection of Methotrexate 15 mg was added to therapy. One month after a full dosage, the steroid was de-escalated by 10% of the initial posology every ten days. In November, after the first two months of treatment, the patient was able to resume a sitting position. CK values decreased slower with statin withdrawal alone (about 3000 U less in about a month) than with the beginning of steroid treatment, whereby CK values have more than halved within 2 weeks. Symptoms also did not regress upon discontinuation of treatment whereas they improved after two months of immunosuppressive treatment.

**Fig. 1:**
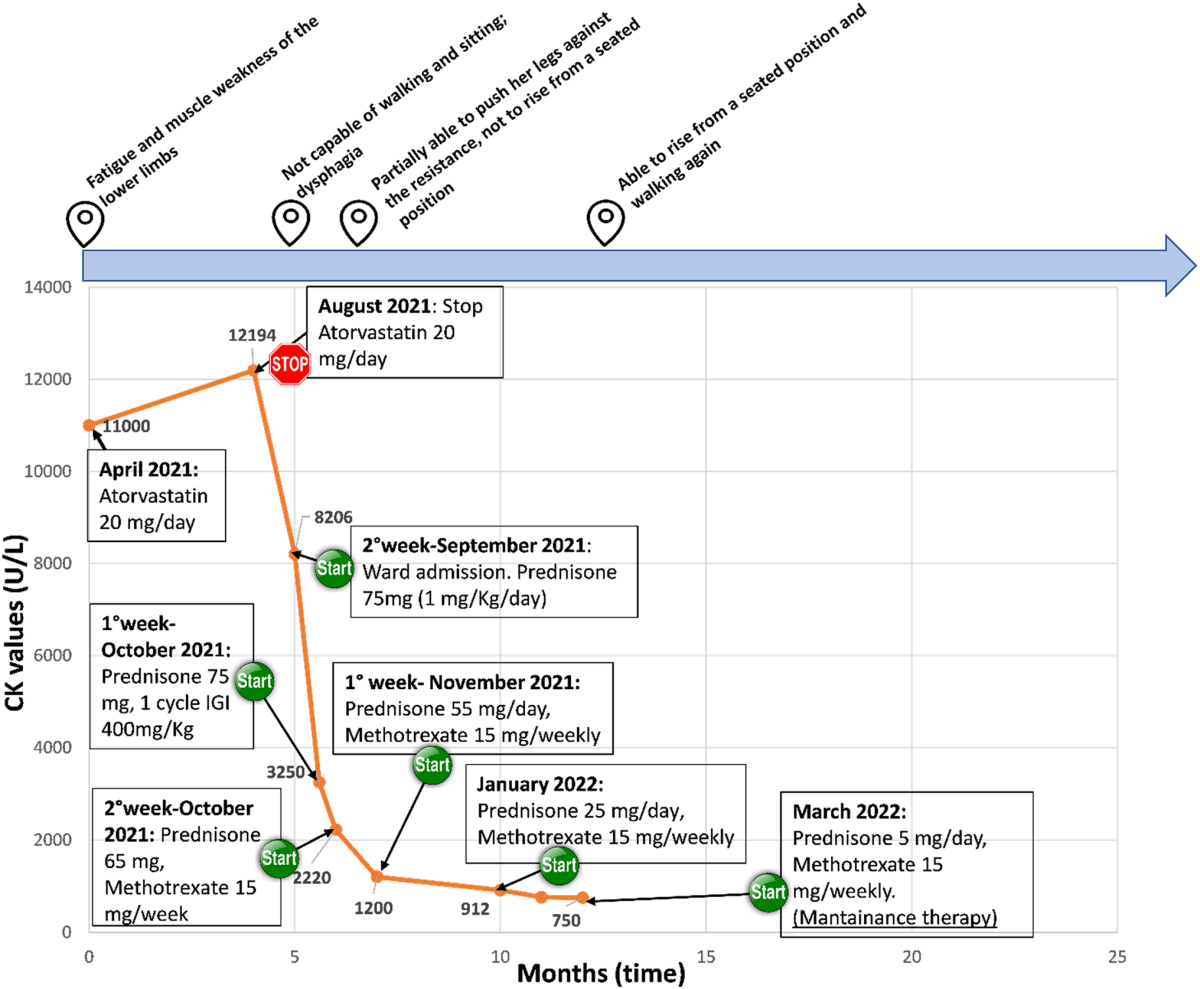
Graph of the time course of CK in relationship with therapy and symptoms. Case 1

In January 2022, after 4 months of therapy, a new musculoskeletal MR of the lower limbs reported a reduction of the hyperintensity of the signal in the middle-distal third of the adductor muscle bilaterally without imbibition of the subcutaneous soft tissues. The EMG/ENG of the upper and lower arms showed myopathic suffering in the process of improvement. As maintenance therapy, since January 2022 prednisone 25 mg/daily and Methotrexate 15 mg/weekly were administered. At the 6 month follow-up, her biochemical exams reported: CK 3 folds the ULN U/L, myoglobin 5 folds the ULN, GOT/GPT and LDH within the normal range. During the follow-up period, the patient underwent a 4 months physical rehabilitation program (since October 2021 to February 2022), at the end of which she started walking again and she denied dysphagia and fatigue in the use of the upper arms (Fig. 1).

Graph of the time course (in months) of CK values (U/L) in relationship with therapy and related timeline of corresponding symptoms. The start and stop symbols indicate when therapy was started or stopped. The long arrow at the top of the figure represents a timeline where the symbol of position corresponds to the precise moment when the indicated symptoms occurred, in parallel with the time course of the CK values. MTX: methotrexate; IVIG: intravenous human immunoglobulin.

#### Case 2

A 67 years-old man with a clinical history of diabetes, hypertension and hypercholesterolemia treated with atorvastatin 20 mg/day for 2 years, in addition to beta-blocker plus sartan therapy to manage high blood pressure. Symptoms started in May 2020 with episodes of accidental falls due to sudden loss of strength in the lower limbs. Successively, he complained of difficulty in climbing stairs and walking long distances. Due to an elevated serum level of CK (36 folds the ULN), statin treatment was stopped. Despite statin discontinuation, CK values slightly decreased, without normalizing. In September 2020 statin therapy was reintroduced, by switching to rosuvastatin. In the following 2 months, he complained of weight loss and progressive difficulty in walking, getting up from a sitting position and loss of strength in the upper limbs. In November 2020 rosuvastatin was stopped and a musculoskeletal MR documented a moderate fibroadipose replacement of the scapular girdle muscles. In the lower limbs, a severe fibroadipose replacement of the large gluteal muscles was reported too, in addition to diffuse proximal muscle oedema of the lower limbs. The EMG/ ENG of the upper and lower limbs showed neurogenic suffering with signs of collateral reinnervation and increased polyphasia of motor unit potentials of all muscles examined. In March 2021 the patient was then referred to a reference in our unit. Serum levels of CK (45,7 folds the ULN) LDH (1,5 folds the ULN) and GOT/GPT (3,5 folds the ULN) were elevated. Negative inflammation markers excluded the infectious etiology of the disorder. The acid alpha-glucosidase enzyme activity test excluded Pompe Disease (dried blood spot-DBS). A second musculoskeletal MR was suggestive of a pattern of necrotizing myositis. The autoimmune serum profile for specific myositis autoantibodies was investigated: ANA, anti-Mi2/alpha and beta nucleosome remodeling and deacetylase complex, anti-SRP, PL7, PL-1, EJ, OJ, Jo 1, PM-Scl100 and PM-Scl75, Ro-52, NXP2, TIF 1g, SAE1 and MDA5 resulted inconclusively. Only a weak positivity was noticed for anti-Ku antibodies, while a significant positivity for the Anti-HMGCR Ab (13 folds the ULN) was documented. Successively, the biopsy of the left vastus lateralis muscle detected a pattern of myopathy with isolated fibers in phagocytosis and motor unit remodeling. Therefore, these evidences defined a necrotizing form of SIAM.

At first, the patient was treated with iv. fluids, prednisone 25 mg/daily and azathioprine 150 mg/daily. Given the lack of response to these treatments, one month later prednisone 25 mg/daily was up titrated to 50 mg/daily and methotrexate 15 mg/weekly was added to the therapy. From June 2021 to February 2022, a monthly cycle of IVIG 400 mg/Kg was administered. Since July 2021 he is still taking methotrexate 15 mg /weekly and prednisone had been de-escalated 10% of the initial posology every 10 days up to 5 mg/daily.

**Fig. 2:**
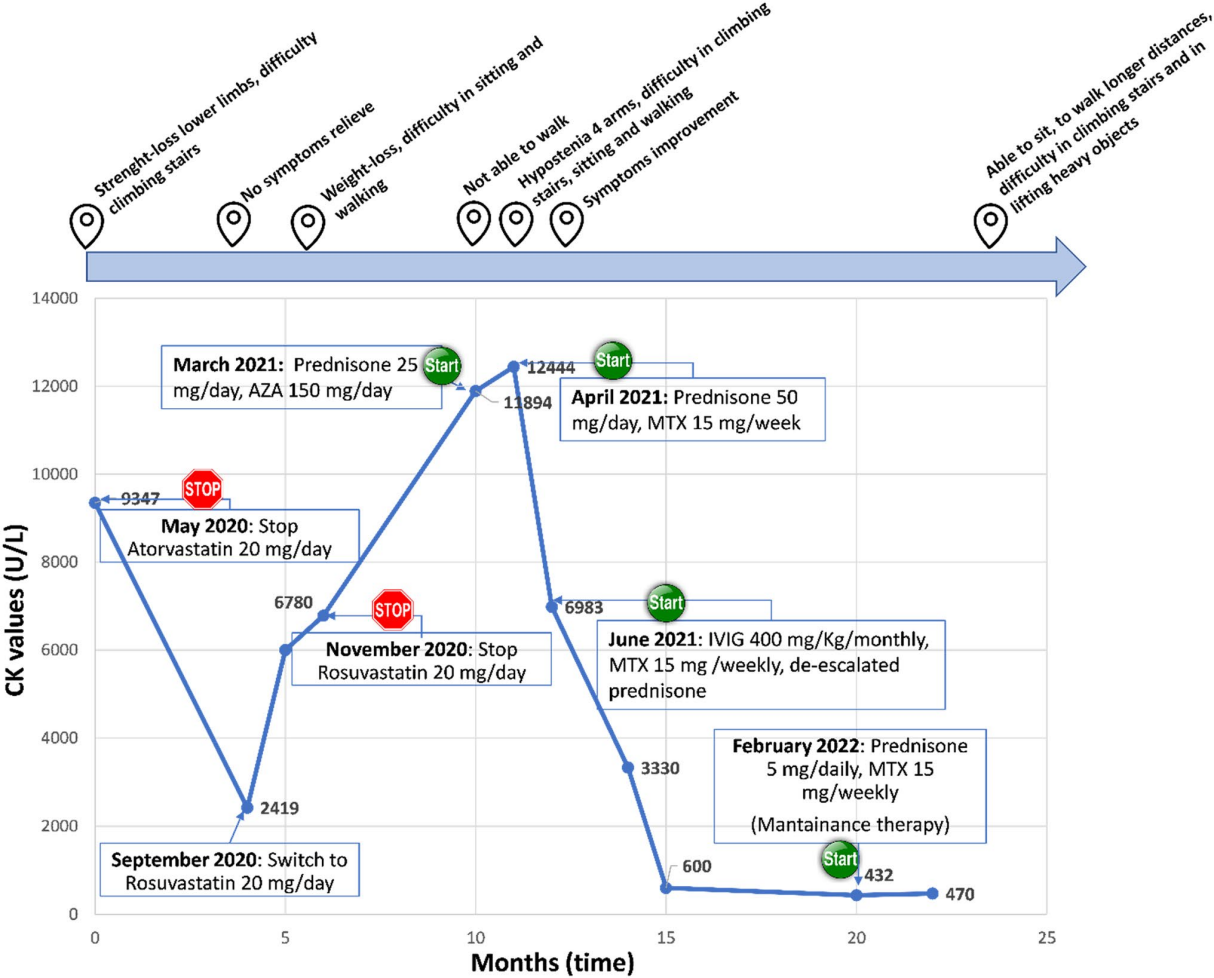
Graph of the time course of CK in relationship with therapy and symptoms. Case 2

At 1 year follow-up the patient reported improvements in maintaining a sitting position and less pain when standing or resting. He still struggles to climb stairs and lift heavy objects. Serum CK has reached stable values around twofolds the ULN (see Fig. 2). Clinical features and the most significant biochemical exams of both patients are detailed in Table 1.

**Table 1:** Main clinical features and serum biochemical exams at the admission ward and at the last follow-up of both patients (6 month follow-up for patient N.1, 1-year follow-up for patient N.2)

Patients	Case N.1		Case N.2	
**Age**	67		67	
**Sex**	Female		Male	
**Race**	Caucasian		Caucasian	
**Comorbidity**				
Hypertension	Yes		No	
Hyperlipidemia	Yes		Yes	
Coronary artery disease	Yes		No	
**Statin treatment and posology**	Atorvastatin 20 mg		Atorvastatin 20 mg; Rosuvastatin 20 mg	
**Comulative time for statin treatment**	10 years		2 years (Atorvastatin); 2 months (Rosuvastatin)	
**Initial symptoms**				
Muscle weakness	Yes		Yes	
Muscle pain	Yes		Yes	
Simmetric distribution	Yes		Yes	
Dysphagia	Yes		No	
Weight-loss	Yes		No	
**Muscle-involvement**				
Proximal muscles	Yes		Yes	
Distal muscles	No		No	
Upper limbs	No		Yes	
Inferior limbs	Yes		Yes	
Muscle atrophy	Yes		No	
Cutaneous rash	Yes		No	
**EMG/ENG**				
Denervation pattern	Yes		Yes	
Re-innervation pattern	No		Yes	
**Musculoskeletal MR**				
Widespread edema	Yes		Yes	
Adipose-sobstitution	No		Yes	
**Biopsy**				
Necrosis pattern	N.A		Yes	
Inflammatory infiltrate	N.A		Yes	
**Antibodies**				
Anti-HMGCR AB	Yes		Yes	
ANA	No		No	
Anti-Ku	Yes		Yes	
**Therapy**				
Steroid	Yes		Yes	
Methotrexate	Yes		Yes	
IntraVenous immune-globulins	Yes		Yes	
Azathioprine	No		Yes	
**SRT in months**	12		24	
**Biochemical Exams**	Entry	Follow-up	Entry	Follow-up
Mioglobin (mc/L) (N.V.: < 58)	4016	296	N.A	N.A
CK (U/L) (N.V.: 192–260)	8206	474	11,894	4687
LDH (U/L) (N.V.: 240–480)	1265	225	713	757
GOT/GPT (U/L) (N.V.: < 31/ < 31)	304/339	26/39	115/138	71/132
Total cholesterol (mg/dl)	200	N.A	N.A	149
LDL cholesterol (mg/dl)	112,8	N.A	N.A	61,2
HDL cholesterol (mg/dl)	38	N.A	N.A	41
Triglycerides (mg/dl)	246	N.A	N.A	234

*EMG/ENG* electromyography/electroneurography, *MR* magnetic resonance, *ANA* anti-nuclear autoantibodies, *SRT* symptoms resolution time, *CK* creatine kinase, *LDL* low-density lipoprotein, *HDL* high-density lipoprotein, *LDH* lactate dehydrogenase, *N.A* not-available

Graph of the time course (in months) of CK values (U/L) in relationship with therapy and related timeline of corresponding symptoms. The start and stop symbols indicate when therapy was started or stopped. The long arrow at the top of the figure represents a timeline where the symbol of position corresponds to the precise moment when the indicated symptoms occurred, in parallel with the time course of the CK values. MTX: methotrexate; IVIG: intravenous human immunoglobulin; AZA: azathioprine.

### Review of published cases

The main clinical features of 69 collected patients are listed in Table 2. The mean age was 63.47 years; female patients were younger than males (61.86 vs 64.73 years). No differences were reported as regards race and comorbidity. Most of the patients assumed atorvastatin as hypolipidemic therapy. The mean cumulative time of statin treatment was 53.04 months (4.41 years). Among the initial symptoms, muscle weakness was referred by most patients, with a symmetric distribution. In the female sex, muscle weakness prevalent in proximal muscles than distal ones (94.2 vs 34.78%, respectively) in both upper and lower limbs and a lower mean value at MRC score were noticed. Muscle pain, dysphagia, neck’s muscles and lower limbs involvement and weight loss were noticed more commonly in males. A denervation pattern at the EMG/ENG was mainly reported in females while in males widespread oedema at MR was more frequent (Table 2). ANA positivity (in addition to anti-HMGCR Ab) was reported only in females. When available, muscle-cell necrosis and inflammatory infiltrate in endomysial and perivascular regions were characteristic features of muscle-biopsy specimens.

**Table 2:** Cumulative data from the selected casuistry

Patients	*M* = 39	*F* = 30	TOT = 69
**Age (mean ± SD)**	64.73 ± 8.35	61.86 ± 9	63.47 ± 8.71
**Race**			
Caucasian	10 (25.64%)	6 (20%)	16 (23.2%)
African	2 (5%)	2 (6%)	4 (5.8%)
African-American	1 (2.5%)	1 (3.33%)	2 (2.9%)
Haitian	0 (0%)	1 (3.33%)	1 (1.45%)
American-Indian	4 (10.25%)	2 (6%)	6 (8.7%)
Indian	2 (5%)	0 (0%)	2 (2.9%)
Hispanic	1 (2,5%)	1 (3.33%)	2 (2.9%)
**Comorbidity**			
Hypertension	27 (69.23%)	20 (66.67%)	47 (68.12%)
Diabetes mellitus 2	19 (48.72%)	14 (46.67%)	33 (47.82%)
Hyperlipidemia	25 (64.1%)	19 (63.33%)	44 (63.76%)
Coronary artery disease	9 (23%)	7 (23.33%)	16 (23.2%)
Stroke	3 (7.7%)	0 (0%)	3 (43.48%)
Chronic kidney disease	2 (5%)	0 (0%)	2 (2.9%)
Neoplasm	1 (2,5%)	1 (3.33%)	2 (2.9%)
Neurological disorders	3 (7.7%)	5 (16.67%)	8 (11.6%)
Reumatological disorders	1 (2.5%)	1 (3.33%)	2 (2.9%)
**Statin treatment**			
Simvastatin	5 (12.8%)	9 (30%)	14 (20.28%)
Atorvastatin	34 (87.18%)	24 (80%)	58 (85.5%)
Rosuvastatin	0 (0%)	1 (3.33%)	1 (1.45%)
Other statins	0 (0%)	2 (6%)	2 (2.9%)
** Comulative time in months (mean ± SD)**	53.83 ± 51.84	51.8 ± 47.25	53.04 ± 49.62
**Initial symptoms**			
Muscle weakness	33 (84.6%)	29 (96.67%)	62 (89.85%)
Stiffness	1 (2.5%)	1 (3.33%)	2 (2.9%)
Muscle pain	11 (28.2%)	4 (13.33%)	15 (21.74%)
Simmetric distribution	36 (92.3%)	29 (96.67%)	65 (94.2%)
Dysphagia	6 (15.38%)	4 (13.33%)	10 (14.5%)
Sistolic heart failure	1 (2,5%)	1 (3.33%)	2 (2.9%)
Dyspnea	4 (10.25%)	3 (10%)	7 (10.14%)
Dark urine	2 (5%)	0 (0%)	2 (2.9%)
Weight-loss	5 (12.8%)	2 (6%)	7 (10.14%)
**Muscle-involvement**			
Proximal muscles	35 (89.74%)	30 (100%)	65 (94.2%)
Distal muscles	14 (35.9%)	10 (33.33%)	24 (34.78%)
Upper limbs	29 (74.34%)	29 (96.67%)	55 (79.71%)
Inferior limbs	34 (87.18%)	29 (96.67%)	63 (91.3%)
Neck	6 (15.38%)	3 (10%)	9 (13.04%)
Minor MRC score (mean ± SD)	3.36 ± 0.95	2.77 ± 1.1	3.07 ± 1.03
Minor MRC score (AV)	1	1	1
Muscle atrophy	2 (5%)	2 (6%)	4 (5.8%)
Cutaneous rash	1 (2.5%)	2 (6%)	3 (4.34%)
**Initial CK value (mean ± SD)**	12,748.63 ± 9956.2	10,201.82 ± 5352	11,616.71 ± 8274.11
**EMG/ENG**	**15/39**	**16/30**	**31/69**
Denervation pattern	13 (86.67%)	16 (100%)	29 (93.55%)
Re-innervation pattern	4 (26.67%)	2 (12.5%)	6 (19.35%)
**Musculoskeletal MR**	**19/39**	**14/30**	**33/69**
Widespread edema	19 (100%)	12 (85.71%)	31 (93.94%)
Adipose-sobstitution	2 (10.53%)	3 (11.5%)	5 (15.15%)
**Biopsy**	**31/39**	**21/30**	**52/69**
Necrosis pattern	31(100%)	21 (100%)	52 (100%)
Inflammatory infiltrate	10 (32.25%)	9 (42,85%)	19 (36.54%)
Lymphocyte infiltrate	2/10 (20%)	1/9 (11%)	3/19 (15,7%)
Macrophages infiltrate	8/10 (80%)	8/9 (89%)	16/19 (84,3%)
**Anti-HMGCR AB**	39 (100%)	30 (100%)	69 (100%)
Anti-SRP AB	0 (0%)	0 (0%)	0 (0%)
ANA	0 (0%)	5 (16.67%)	5 (7.24%)
Anti-Ro	1 (2,5%)	1 (3.33%)	2 (2.9%)
Anti-Ku	1 (2,5%)	2 (6%)	3 (4.34%)
Anti- SCL7	0 (0%)	1 (3.33%)	1 (1.45%)
Anti-Sm	1 (2,5%)	0 (0%)	1 (1.45%)
Anti-MDA 5	0 (0%)	1 (3.33%)	1 (1.45%)
**Therapy**			
Steroid	33 (84.6%)	27 (90%)	60 (86.95%)
Methotrexate	12 (30.77%)	17 (56.67%)	29 (42.02%)
IntraVenous Immune-Globulins	25 (64.1%)	15 (50%)	40 (57.97%)
Azathioprine	8 (20.51%)	4 (13.33%)	12 (17.39%)
Cyclophosphamide	4 (10.25%)	2 (6%)	6 (8.7%)
Mycophenolate	5 (12.8%)	2 (6%)	7 (10.14%)
Rituximab	8 (20.51%)	7 (23.33%)	15 (21.74%)
Tacrolimus	0 (0%)	1 (3.33%)	1 (1.45%)
**SRT in months (mean ± SD)**	7.88 ± 9.48	16.65 ± 28.29	12.14 ± 20.47
**Last CK value (mean ± SD)**	1440.5 ± 1383.39	312.81 ± 325.32	688.7 ± 972.81

Results from the frequency and descriptive statistical analysis in 69 patients collected from literature (67 patients) and our case records (2 patients)

*M* male, *F* female, *TOT* total, *SD* standard deviation, *MRC* Medical Research Council scale for Muscle Strength (0: no contraction, 1 Flicker or trace of contraction, 2 Active movement, with gravity eliminated, 3 Active movement against gravity, 4 Active movement against gravity and resistance, 5 Normal power), *AV* absolute value, *ANA* anti-nuclear autoantibodies, *CK* creatine kinase, *SRT* symptoms resolution time

### Diagnostic algorithm

**Fig. 3:**
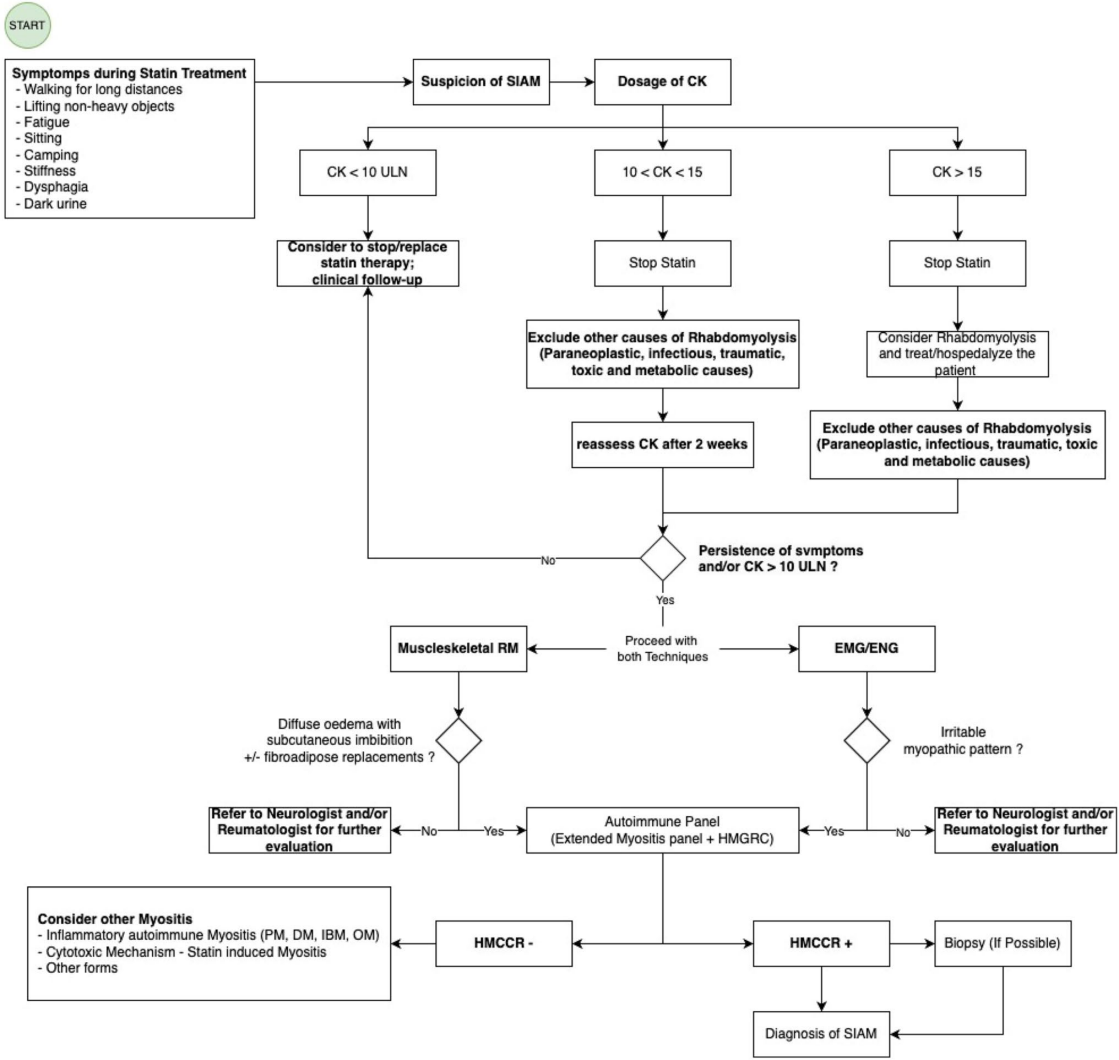
Diagnostic flow-chart of SIAM

The algorithm entry point is represented by any of the symptoms occurring during prolonged statin therapy, that should induce the clinical suspicion of SIAM (Fig. 3). The term “prolonged” identifies a period of continuous statin intake of more than 6 months. If SIAM is suspected, a CK evaluation must be performed. CK values ≥ 10 folds the ULN are suggestive of SIAM and statin treatment must be discontinued. [8]. If CK values > 15 folds the ULN, associated with myoglobinuria and acute kidney injury, rhabdomyolysis must be considered and treated (consider to hospitalize the patient). Other causes of rhabdomyolysis must be excluded: paraneoplastic, infectious, traumatic, toxic and metabolic causes [13]. After 2 weeks from statin discontinuation, dosage of CK should be repeated: values < 10 folds the ULN should be followed-up, without continuing with second-level tests. If symptoms do not relieve and CK values persist to be elevated > 10 folds the ULN after 2 weeks, an EMG/ ENG and a musculoskeletal MR should be performed. A suggestive EMG/ ENG must report sign of irritable myopathy, while a typical MR has a diffuse oedema and subcutaneous imbibition, with or without signs of fibroadipose replacement. If one or both tests do not reveal these findings, the patient should be referred to the neurologist or rheumatologist for further evaluation. Conversely, in presence of evocative EMG/ENG and/or musculoskeletal MR, the algorithm gives an indication for assays of anti-SRP, anti-HMGCR Ab and the autoimmune panel (including the extended Myositis panel anti-Mi2/alpha and beta, anti-DNA, Ku, SRP, PL7, PL-12, EJ, OJ, Jo 1, PM-Scl100 and PM-Sc75, Ro-52, NXP2, TIF 1g, SAE1 and MDA5). A positive test for anti-HMGCR (tested with ELISA assay) Ab defines SIAM, while a negative result orients towards other forms of autoimmune myositis (the autoimmune panel contributes to define alternative diagnoses). Then, a muscle biopsy can support the diagnosis, although it is not mandatory to make the diagnosis of SIAM. A muscle biopsy can identify the necrotizing pattern and/or the presence of inflammatory infiltrates that may contribute to define the disease severity. The algorithm is described in the flowchart of Fig. 3.

Flowchart of our proposed algorithm for the diagnosis of Statin-induced autoimmune myositis (SIAM). This algorithm starts (follows the draw) from the recognition of symptoms occurred during the statin treatment, indicated in the specific box. Once SIAM is suspected, creatin-kinase (CK) dosage must be performed. If CK values maintain < 10 folds the upper limit of normal (ULN), the clinician should consider to stop/replace statin treatment and continue the follow-up. Values > 10 folds the ULN are suggestive of SIAM and statin therapy must be stopped as soon as possible. If CK values > 15 folds the ULN, associated with myoglobinuria and acute kidney injury, rhabdomyolysis must be considered and treated (consider to hospitalize the patient). Regardless, other causes of Rhabdomyolysis must be excluded: paraneoplastic, infectious, traumatic, toxic and metabolic causes. After 2 weeks from statin discontinuation, a dosage of CK should be repeated: values under 10 folds the ULN should be followed-up, without continuing with second-level tests. If symptoms do not relieve and CK values persist to be elevated > 10 folds the ULN after 2 weeks, an electromyography/electroneurography (EMG/ ENG) and a musculoskeletal MR should be performed. A suggestive EMG/ENG shows sign of irritable myopathy, while a typical MR a diffuse oedema and subcutaneous imbibition, with or without signs of fibroadipose replacement. If one or both of these test result inconclusive, the patient should be referred to the neurologist or rheumatologist for further evaluation. If suggestive findings are identified at the EMG/ ENG and/or musculoskeletal MR, the algorithm provides for the autoimmune panel, including the extended Myositis panel (anti-Mi2/alpha and beta, anti-DNA, Ku, SRP, PL7, PL-12, EJ, OJ, Jo 1, PM-Scl100 and PM-Sc75, Ro-52, NXP2, TIF 1g, SAE1 and MDA5), anti-SRP and anti-HMGCR Ab. Positivity of anti-HMGCR defines SIAM, conversely another form of autoimmune Myositis should be considered (the autoimmune panel detects the specific Myositis pattern). Then, a muscle biopsy can establish the diagnosis of certain, although it is not necessary to reach the clinical diagnosis of SIAM. Biopsy can identify the necrotizing pattern and/or inflammatory infiltrates that contribute to define the disease severity. Immunohistochemistry must be applied to muscle specimens.

## Discussion

SIMS defines a heterogeneous group of clinical presentations which occur as rare adverse events after the statin treatment. The criteria of the American College of Cardiology (ACC), the American Heart Association (AHA) and the National Heart, Lung, and Blood Institute (NHLBI) describe three types of SIM [14]: (i) Statin-induced myalgia: muscle symptoms without CK elevations, (ii) Statin-induced myositis: muscle symptoms with CK elevations., (iii) Statin-induced rhabdomyolysis: muscle symptoms with marked CK elevations (over ten times the ULN) with an elevated creatinine value and the occasional presence of brown urine (myoglobinuria). Statin-induced myositis recognizes at first a most common cytotoxic mechanism; less frequently an autoimmune mechanism. The finding of anti-HMGCR Ab defines SIAM, that is considered a quite different clinical entity than statin-induced myositis. Whether SIAM represents a more severe clinical presentation of statin-induced myositis, or it should be considered a subtype of autoimmune myositis, remains a topic of scientific debate [7]. The autoimmune mechanism should be suspected over the more common cytotoxic mechanism at the persistence of symptoms, even after discontinuation of therapy [6]. As shown by the algorithm, HMGCR-ab negativity in the face of statin therapy should suggest other forms of myositis including statin-induced forms with cytotoxic mechanism.

SIAM onset can be acute (days to weeks) or sub-acute (< 6 months). Generally, symptoms occur after a prolonged statin treatment and their resolution can be very slow, even after several months. The term “prolonged” identifies a period of continuous statin intake of more than 6 months. This period was assessed by observing data extrapolated from the selected articles, in which the shortest time of statin treatment before symptoms was around 8 months. The definition of 6 months as "prolonged use of statin" is also consistent with other publications regarding SIAM [15]. The broad spectrum of clinical manifestations of our patients’ cohort accurately depicts SIAM clinic [16]. The algorithm we propose starts from the recognition of the symptoms during statin therapy. Symmetric-proximal muscle weakness was referred to as the most common initial symptom in our cohort.

Atorvastatin resulted the statin being more associated with SIAM in the case series we examined. Also simvastatin played a relevant role in SIAM, although prevalence rates were 20.28 vs 85.5% of atorvastatin, respectively (Table 2). Although a formal statistical analysis was not possible with the collected data, these suggest a more frequent occurrence of SIAM with atorvastatin, than with other statins. Simvastatin and atorvastatin have a predominantly hepatic metabolism and are metabolized by cytochrome CYP3A4, releasing their metabolites into plasma. This metabolic pathway lends itself to multiple interactions with drugs metabolized by the CYP3A4 pathway. Simvastatin and Atorvastatin also have greater lipophilic properties and penetrate peripheral tissue cells better as well as liver cells. Rosuvastatin, along with fluvastatin and others, is only partially metabolized via the cytochrome CYP2C9 pathway. Rosuvastatin is excreted both via bile and urea and also exhibits hydrophilic properties. These chemical characteristics result in a reduced penetration of Rosuvastatin into peripheral tissues [16].

The Medical Research Council (MRC) scale for Muscle Strength represents the most frequent adopted criterion to clinically describe the grade of muscle affection (0: no contraction; 1: flicker or trace of contraction; 2: active movement, with gravity eliminated; 3: active movement against gravity; 4: active movement against gravity and resistance; 5: normal power) [18]. Lower values at the MRC score have been noticed in females than males (2.77 vs 3.36, respectively); in addition, a recurrent denervation pattern at the EMG/ENG, adipose substitution at the MR, presence of ANA Ab (in addition to anti-HMGCR Ab) and larger inflammatory infiltrates at biopsy suggest a more severe disease in female patients. Women notoriously have a predisposition towards autoimmunity and it probably gives them an additional risk factor to develop SIAM and/or to present a more severe disease.

Once SIAM is suspected, CK assay is mandatory. Values > 10 folds the ULN are suggestive of SIAM and statin therapy must be stopped as soon as possible. As an addition to the only available proposed algorithm [8], we propose a shorter interval between the first CK assay and the second evaluation (2 vs 8 weeks) with the aim to anticipate the suitable treatment as soon as possible. CK values > 15 folds the ULN in association with acute symptoms are suggestive of rhabdomyolysis, so our algorithm proposes to directly treat/hospitalize the patient. If the CK < 10 the ULN, the algorithm recommends follow-up. The algorithm considers an individualized and patient-tailored management in terms of timing, and follow-up schedule. The algorithm also emphasizes the exclusion of other causes of rhabdomyolysis (paraneoplastic, infectious, traumatic, toxic and metabolic causes).

In addition, we propose to add the EMG/ENG and musculoskeletal MRI to the diagnostic workup before proceeding with the antibody assay. These tests allow us to document the organ damage of myositis and contribute to define the severity of the organ damage itself. EMG/ENG is altered in different forms of myositis and shows signs of irritable myopathic pattern with signs of denervation [19]. EMG/ENG also makes it possible to differentiate inflammatory forms of myositis from other forms of muscle weakness, including weakness associated with steroid use. However, EMG/ENG does not allow differentiation between the subtypes of myositis. It is also useful for evaluating 'acute' versus chronic forms of myopathies [20].

MRI with STIR sequences has a sensitivity of 89–100% for detecting inflammatory changes, which is higher than muscle biopsy at 66% [21, 22], while MRI specificity is estimated around the 80–88% against the specificity of 100% for muscle biopsy [23]. The musculoskeletal MR of the involved limbs typically shows symmetrical and diffuse intramuscular and intrafascial oedema in anterior, medial and posterior compartments of the affected muscles. In addition to oedema, other pathological changes may occur within the muscle as a consequence of the chronic inflammatory myopathic process, including loss of muscle bulk (atrophy), fatty involution or replacement with connective tissue. The necrotizing form of SIAM is characterized by more widespread muscle involvement and signs of fibroadipose substitution [21]. However, it must be emphasized that MRI alone cannot discriminate between the various forms of myositis but needs to be corroborated by the other clinical elements (symptoms, ck, autoantibodies, etc.).

If EMG/ ENG and/or musculoskeletal MR are compatible with SIAM, the next step consists of the analysis of autoantibodies patterns, including the extended myositis panel (anti-Mi2/alpha and beta, anti-DNA, Ku, SRP, PL7, PL-12, EJ, OJ, Jo 1, PM-Scl100 and PM-Sc75, Ro-52, NXP2, TIF 1g, SAE1 and MDA5) and anti-HMGCR Ab, necessary to make a differential diagnosis between SIAM and other forms of autoimmune myositis [24]. Anti-HMGCR Ab always results negatively in Polymyositis (PM), Dermatomyositis (DM) and Inclusion body Myositis (IBM) [25]. Necrotizing myositis (paraneoplastic syndrome, drugs, toxicity and other) can mime SIAM, showing the presence of anti-SRP Ab and/or anti-HMGCR Ab regardless of the absence of statin exposure [10–23].

ANA are usually detected in PM, DM and IBM but their positivity is inconclusive in SIAM. Among the described cases, ANA positivity was found only in 5 females out of 69 patients. An uncommon finding is a weak anti-Ku positivity. This was found in both cases described in this report and in one additional case previously described in which ANA were also positive [26]. Currently, no clinical significance can be attributed to this finding.

Muscle biopsy is the last step to support the diagnosis Biopsy is able to assess the various forms of inflammatory myositis and to distinguish these from necrotizing forms. It also permits to assess of the degree of severity of the pathology by highlighting the type of inflammatory infiltrate. Being biopsy invasive and not always easy to perform, it becomes useful to confirm the diagnosis and it is reserved for doubtful cases [20]. Cellular infiltrate is composed largely of macrophages over the CD4+ and CD8+ lymphocyte population (macrophages probably play a role in tissue repair). The hematoxylin and eosin staining of a paraffin-embedded specimen reveals abundant myophagocytosis [27]. Diffuse or multifocal up-regulation of MHC I molecules is common. Nevertheless, sarcolemmal MHC I staining appears to be particularly specific to SIAM, such as staining is only rarely noted in metabolic or genetic muscle disorders [28].

In some patients, muscle weakness persisted even after the muscle enzyme levels have returned within the normal range [27]. Symptoms resolution time (SRT) had required a mean duration of 12.14 months, longer in females (16.65 months) than in males (7.88 months). The last one data confirms the suggestion of more severe disease in females than in the male group.

Once few consensus and/or diagnostic criteria for myositis have been assessed and only a single diagnostic algorithm specific for SIAM has been published in 2016 [8]. Moreover, given the continuous modernization of imaging/instrumental techniques, few diagnostic criteria for myositis take into account them. For example, the MRI finding of oedema on STIR imaging has been inserted as one of the variables of the European Neuro-Muscular Centre classification criteria from myositis of 2004 [29] but it has been recently excluded by the diagnostic criteria of the International Myositis Evaluation and Clinical Trials Group (IMACS) of 2016, as MRI was not widely used in the study population from which the criteria variables were derived [30]. Our algorithm is also updated to include the most modern diagnostic/imaging techniques in the diagnostic pattern-.

However, to date the study presents many limits. Once the study is not designed as a ‘systematic review’ of the literature, the methodological quality of the work was not assessed and we have not applied statistical analyses as we disposed only data extrapolated by articles but not the raw data. In fact, this does not allow us to provide clear associations/correlations between the various clinical elements of SIAM examined to date. Moreover, this work does not deal with the topic of SIAM therapy. The reviewed data did not allow to make a clear statement on treatment options. Further works have to be done to provide treatment guidelines to the medical community.

In conclusion, we think that our “experience-based algorithm” can provide a modern and flexible tool to early achieve a diagnosis of SIAM as specific as possible, adding a relevant contribution to the current clinical practice in the management of SIAM.

The different clinical presentations of SIAM, and the diagnostic-therapeutic approaches described in the various studies that we have reviewed, provide the scientific community a clarifying contribution to this not fully acknowledged clinical entity. As a final consideration, the take-home message should be to monitor closely any symptoms occurring during statin treatment so as to recognize and treat adverse events as early as possible.

## Supplementary Information

Below is the link to the electronic supplementary material.

Supplementary file1 (DOCX 125 KB)

## Data Availability

Data sharing not applicable to this article as no datasets were generated or analysed during the current study. Supplementary informations are provided in the Supplementary electronic file.

## References

[1] Mammen AL, Chung T, Christopher-Stine L, Rosen P, Rosen A, Doering KR (2011). Autoantibodies against 3-hydroxy-3-methylglutaryl-coenzyme A reductase in patients with statin associated autoimmune myopathy. Arthritis Rheumatism.

[2] Hamann PD, Cooper RG, McHugh NJ, Chinoy H (2013). Statin-induced necrotizing myositis—a discrete autoimmune entity within the “statin-induced myopathy spectrum”. Autoimmun Rev.

[3] Josan K, Majumdar SR, McAlister FA (2008). The efficacy and safety of intensive statin therapy: a meta-analysis of randomized trials. CMAJ.

[4] Christopher-Stine L, Casciola-Rosen LA, Hong G, Chung T, Corse AM, Mammen AL (2010). A novel autoantibody recognizing 200-kd and 100-kd proteins is associated with an immune-mediated necrotizing myopathy. Arthritis Rheum.

[5] Nazir S, Lohani S, Tachamo N, Poudel D, Donato N (2017). Statin-induced autoimmune myopathy: a systematic review of 100 cases. J Clin Rheumatol.

[6] Freeman MW, Singh AK, Guidon AC, Arvikar SL, Goldstein RH, Clement NF (2019). Case 22–2019: a 65-year-old woman with weakness, dark urine, and dysphagia. N Engl J Med.

[7] Jayatilaka S, Desai K, Rijal S, ZimmeMRan D (2021). Statin-induced autoimmune necrotizing myopathy. J Prim Care Community Health.

[8] Mammen AL (2016). Statin-associated autoimmune myopathy. N Engl J Med.

[9] Cervellin G, Comelli I, Benatti M, Sanchis-Gomar F, Bassi A, Lippi G (2017). Non-traumatic rhabdomyolysis: background, laboratory features, and acute clinical management. Clin Biochem.

[10] Schmidt J (2018). Current classification and management of inflammatory myopathies. J Neuromuscul Dis.

[11] Dankó K, Ponyi A, Molnar AP, András C, Constantin T (2009). Paraneoplastic myopathy. Curr Opin Rheumatol.

[12] Maheswaranathan M, Johannemann A, Weiner JJ, Jessee R, Eudy AM, Criscione-Schreiber L (2021). Real world utilization of the myositis autoantibody panel. Clin Rheumatol.

[13] Melli G, Chaudhry V, Cornblath DR (2005). Rhabdomyolysis: an evaluation of 475 hospitalized patients. Medicine (Baltimore).

[14] Pasternak RC, Smith SC, Bairey-Merz CN, Grundy SM, Cleeman JI, Lenfant C (2002) ACC/AHA/NHLBI clinical advisory on the use and safety of statins12. J Am Coll Cardiol Am Coll Cardio Found 40(3):567–72 10.1016/s0735-1097(02)02030-2 12142128

[15] Hansen KE, Hildebrand JP, Ferguson EE, Stein JH (2005). Outcomes in 45 patients with statin-associated myopathy. Arch Intern Med.

[16] Meyer A, Troyanov Y, Drouin J, Oligny-Longpré G, Landon-Cardinal O, Hoa S (2020). Statin-induced anti-HMGCR myopathy: successful therapeutic strategies for corticosteroid-free remission in 55 patients. Arthritis Res Ther.

[17] Neuvonen PJ, Niemi M, Backman JT (2006). Drug interactions with lipid-lowering drugs: mechanisms and clinical relevance. Clin Pharmacol Ther.

[18] Vanhoutte EK, Faber CH,1 Van Nes SI, Jacobs BC, Van Doorn PA, Van Koningsveld R et al (2012). Modifying the medical research council grading system through Rasch analyses. Brain. 135(5): 1639–1649 10.1093/brain/awr318 PMC3338921 22189568

[19] Karunaratne K, Amiras D, Pickering MC, Hofer M, Viegas S (2018). Autoimmune necrotising myopathy and HMGCR antibodies. Pract Neurol.

[20] Carstens PO, Schmidt J (2014). Diagnosis, pathogenesis and treatment of myositis: recent advances. Clin Exp Immunol.

[21] Day J, Patel S, Limaye V (2017). The role of magnetic resonance imaging techniques in evaluation and management of the idiopathic inflammatory myopathies. Semin Arthritis Rheum.

[22] Badrising UA, Kan H, Verschuuren JJ, Weber MA (2013). MRI in inflammatory myopathies and autoimmune-mediated myositis. Magnetic resonance imaging of the skeletal musculature.

[23] Dion E, Cherin P, Payan C, Fournet J-C, Papo T, Maisonobe T (2002). Magnetic resonance imaging criteria for distinguishing between inclusion body myositis and polymyositis. J Rheumatol.

[24] Troyanov Y, Landon-Cardinal O, Fritzler MJ, Ferreira J, Targoff IN, Rich E (2017). Atorvastatin-induced necrotizing autoimmune myositis: an emerging dominant entity in patients with autoimmune myositis presenting with a pure polymyositis phenotype. Medicine (Baltimore).

[25] Alzueta N, Marin M, Castresana M, Gascón A, Pío M, Iguzquiza MJ (2021). Statin-induced autoimmune myopathy: a case report. Eur J Hosp Pharm.

[26] Mishouri P, Prodip P, Dipon D, Syed WM, Fariya S (2021). A case of statin-associated immune-mediated necrotizing myopathy, successfully treated with intravenous immunoglobulin. Cureus.

[27] Stenzel W, Goebel HH, Aronica E (2012). Review: immune-mediated necrotizing myopathies—a heterogeneous group of diseases with specific myopathological features. Neuropathol Appl Neurobiol.

[28] SEARCH Collaborative Group. Link E, Parish S, Armitage J, Bowman L, Heath S, et al (2008) SLCO1B1 variants and statin-induced myopathy—a genomewide study. N Engl J Med 359(8):789–799 10.1056/NEJMoa0801936 18650507

[29] Hoogendijk JE, Amato AA, Lecky BR, Choy EH, Lundberg IE, Rose MR (2004). 119th ENMC international workshop: trial design in adult idiopathic inflammatory myopathies, with the exception of inclusion body myositis, Naarden, The Netherlands. Neuromuscul Disord.

[30] Lundberg IE, Miller FW, Tjärnlund A, Bottai A (2016). Diagnosis and classification of idiopathic inflammatory myopathies. J Intern Med.

