# Supplementary material for: Statin-induced autoimmune myositis: a proposal of an “experience-based” diagnostic algorithm from the analysis of 69 patients
Source: Intern Emerg Med. 2023 May 5;18(4):1095–107. doi: 10.1007/s11739-023-03278-9 (PMC10326147; doi:10.1007/s11739-023-03278-9)
Supplement: Supplementary file 1 — Supplementary file1 (DOCX 125 KB) [file 11739_2023_3278_MOESM1_ESM.docx]

**Statin-induced autoimmune Myositis: a proposal of an “****experience-based” diagnostic algorithm from the analysis of 69 patients**

**Carola Maria Gagliardo^1^*, Davide Noto^1^*^ǂ^, Antonina Giammanco^1^, Silvia Maltese^1^, Luca Vecchio^1^, Giuseppe Lavatura^1^, Valentina Cacciatore^2^, Carlo Maria Barbagallo^1^, Antonina Ganci^1^, Emilio Nardi^1^, Rosalia Lo Presti^3^, Angelo Baldassare Cefalù**^1^, Maurizio Averna**^1^**

**SUPPLEMENTARY MATERIAL**

**Figure 1. Flow-chart for articles selecting criteria.**

**Table 1. References of the articles from which the data of the 67 collected patients have been Extrapolated**

**Table 2. Serum complete biochemical exams of the patients presented in our two case reports**

**Figure 1. Flow-chart for articles selecting criteria.**

**^^**

**^E TAGLIATO a DESTRA^**

Flow chart regarding the selction criteria for articles eligibility. Literature search was carried out on PubMed, examining case records regarding SIAM. The search terms were: “Statin-induced autoimmune Myopathy Case report”, “Statin-induced autoimmune Myositis Case report”, “HMGCR myopathy Case report”, “HMGCR myositis Case report”. Once these keywords were searched, a total of 191 case reports concerning SIAM were identified and duplicates were excluded (total articles remaining:121). Of the remaining articles, first only english-language articles were selected (17 articles were excluded) and then only full-available articles were selected (15 articles were further excluded). Of the remaining articles (89), a total of 55 articles were selected according to the following inclusion criteria: articles as complete as possible with information on the patients' diagnostic *iter* and concerning only statin-induced forms of myositis with an autoimmune mechanism. A total number of 34 articles that were not complete with various clinical information of the patients, concerning statin-induced forms of myositis with cytotoxic mechanism and/or HMGCR-ab positive but paraneoplastic forms of myositis were excluded.

**Table 1. References** **of the articles from which the data of the 67 collected patients have been Extrapolated**

| **REFERENCES** | **PMID** | **PMCID** |
| --- | --- | --- |
| 1. Lahaye C, Beaufrére AM, Boyer O, Drouot L, Soubrier M, Tournadre A. Immune-mediated myopathy related to anti 3-hydroxy-3-methylglutaryl-coenzyme A reductase antibodies as an emerging cause of necrotizing myopathy induced by statins. Joint Bone Spine. 2014 Jan;81(1):79-82. doi: 10.1016/j.jbspin.2013.06.008 | 23953224 | NA |
| 1. Mygland Å, Ljøstad U, Krossnes BK. Persisting weakness after withdrawal of a statin. BMJ Case Rep. 2014 Apr 8;2014:bcr2013203094. doi: 10.1136/bcr-2013-203094. | 24713712 | PMC3987559. |
| 1. Young JB, Ghobrial II. Autoimmune statin-induced myopathy: a case report. J Community Hosp Intern Med Perspect. 2015 Sep 1;5(4):28374. doi: 10.3402/jchimp.v5.28374. | 26333863 | PMC4558286. |
| 1. Nichols L, Pfeifer K, Mammen AL, Shahnoor N, Konersman CG. An Unusual Case of Statin-Induced Myopathy: Anti-HMGCoA Necrotizing Autoimmune Myopathy. J Gen Intern Med. 2015 Dec;30(12):1879-83. doi: 10.1007/s11606-015-3303-9. | 25855481 | PMC4636568. |
| 1. Ngo LQ, Wu AG, Nguyen MA, McPherson LE, Gertner E. A case report of autoimmune necrotizing myositis presenting as dysphagia and neck swelling. BMC Ear Nose Throat Disord. 2016 May 17;16:7. d | 27190496 | PMC4869331. |
| 1. McGrath NM, Turner CP. Isolated gluteal and paravertebral muscle weakness due to anti-3-hydroxy-3-methylglutaryl-coenzyme a reductase antibody-associated necrotizing autoimmune myopathy. Muscle Nerve. 2016 Jun;54(1):150-152. doi: 10.1002/mus.25130. | 27060972 | NA |
| 1. Hussenbux A, Hofer M, Steuer A. Statin-induced necrotizing autoimmune myopathy: importance of early recognition. Br J Hosp Med (Lond). 2017 Jun 2;78(6):352-353. doi: 10.12968/hmed.2017.78.6.352. | 28614015 | NA |
| 1. Sweidan AJ, Leung A, Kaiser CJ, Strube SJ, Dokukin AN, Romansky S, Farjami S. A Case of Statin-Associated Autoimmune Myopathy. Clin Med Insights Case Rep. 2017 Mar 30;10:1179547616688231. doi: 10.1177/1179547616688231. | 28469499 | PMC5398416 |
| 1. Kunwar S, Parekh JD, Chilukuri RS, Andukuri VA. Necrotizing Autoimmune myopathy: A case report on statin induced rhabdomyolysis requiring immunosuppressive therapy. Drug Discov Ther. 2018;12(5):315-317. doi: 10.5582/ddt.2018.01049 | 30464165 | NA |
| 1. Dixit A, Abrudescu A. A Case of Atorvastatin-Associated Necrotizing Autoimmune Myopathy, Mimicking Idiopathic Polymyositis. Case Rep Rheumatol. 2018 Jun 20;2018:5931046. doi: 10.1155/2018/5931046. | 30026996 | PMC6031080 |
| 1. De Cock E, Hannon H, Moerman V, Schurgers M. Statin-induced myopathy: a case report. Eur Heart J Case Rep. 2018 Nov 27;2(4):yty130. doi: 10.1093/ehjcr/yty130. | 31020206 | PMC6426022 |
| 1. Karunaratne K, Amiras D, Pickering MC, Hofer M, Viegas S. Autoimmune necrotising myopathy and HMGCR antibodies. Pract Neurol. 2018 Apr;18(2):151-155. doi: 10.1136/practneurol-2017-001848. | 29439058 | NA |
| 1. Carvalho AAS, da Silva VG, Zanoteli E, Feder D. Myopathy due to HMGCR antibodies in adult mimicking muscular dystrophy associated with cancer and statin exposure - narrative review of the literature - case report. Ther Clin Risk Manag. 2018 May 14;14:903-907. doi: 10.2147/TCRM.S162931 | 29785116 | PMC5957055 |
| 1. Abdilla Y, Chircop C, Vella N. Anti-HMGCR antibody-associated necrotising myopathy and its association with statin use. BMJ Case Rep. 2018 Nov 12;2018:bcr2018226302. doi: 10.1136/bcr-2018-226302. | 30420560 | PMC6254459 |
| 1. Liang E, Rastegar M. Immune-mediated necrotising myopathy: a rare cause of hyperCKaemia. BMJ Case Rep. 2018 Apr 24;2018:bcr2017223870. doi: 10.1136/bcr-2017-223870 | 29691272 | PMC5926624 |
| 1. Upreti S, Fayyaz B, Bongu RP. Anti-HMG-CoA reductase myopathy, an undesirable evolution of statin induced myopathy: a case report. J Community Hosp Intern Med Perspect. 2019 Feb 11;9(1):33-35. doi: 10.1080/20009666.2019.1571882 | 30788073 | PMC6374921 |
| 1. Saleh Y, Herzallah K, Hassanein M, Chang HT. Statin-induced necrotizing autoimmune myopathy: An uncommon complication of a commonly used medication. J Saudi Heart Assoc. 2019 Oct;31(4):269-272. doi: 10.1016/j.jsha.2019.08.001 | 31516306 | PMC6727174 |
| 1. Sharma P, Timilsina B, Adhikari J, Parajuli P, Dhital R, Tachamo N. Statin-induced necrotizing autoimmune myopathy: an extremely rare adverse effect from statin use. J Community Hosp Intern Med Perspect. 2019 Dec 14;9(6):503-506. doi: 10.1080/20009666.2019.1702272. | 32002159 | PMC6968631 |
| 1. Pitlick M, Ernste F. Anti-HMGCR myopathy presenting with acute systolic heart failure. BMJ Case Rep. 2019 May 8;12(5):e230213. doi: 10.1136/bcr-2019-230213 | 31068355 | PMC6506079 |
| 1. Zhang W, Prince HM, Reardon K. Statin-induced anti-HMGCR antibody-related immune-mediated necrotising myositis achieving complete remission with rituximab. BMJ Case Rep. 2019 Dec 1;12(11):e232406. doi: 10.1136/bcr-2019-232406. | 31791994 | PMC6887445 |
| 1. Shuster S, Awad S. A RARE CASE OF STATIN-INDUCED NECROTIZING AUTOIMMUNE MYOPATHY. AACE Clin Case Rep. 2020 Jan 22;6(2):e86-e89. doi: 10.4158/ACCR-2019-0547 | 32524017 | PMC7282149 |
| 1. Gawey B, Tannu M, Rim J, Sperling L, Henry TL. Statin-Induced Necrotizing Autoimmune Myopathy. JACC Case Rep. 2020 Feb 26;2(3):440-443. doi: 10.1016/j.jaccas.2019.12.019. | 34317259 | PMC8311592 |
| 1. Ahmed S, Capric V, Khan M, Koneru P. A Rapidly Progressive Case of Statin-induced Necrotizing Autoimmune Myopathy. Cureus. 2020 Feb 17;12(2):e7021. doi: 10.7759/cureus.7021 | 32211257 | PMC7081733 |
| 1. Huda SA, Yadava S, Kahlown S, Farooqi MS, Bryant S, Russo R. Statin-induced necrotizing autoimmune myopathy. Proc (Bayl Univ Med Cent). 2020 Oct 21;34(1):185-186. doi: 10.1080/08998280.2020.1831298. | 33456195 | PMC7785182 |
| 1. Madgula AS, Gadela NV, Singh M, Chen K. A Rare Case of Statin-induced Immune-mediated Necrotizing Myopathy. Cureus. 2020 Apr 1;12(4):e7500. doi: 10.7759/cureus.7500. | 32373404 | PMC7195195 |
| 1. Lempel M, Molla E. Treatment of Statin-Induced Necrotizing Autoimmune Myopathy With Glucocorticoid Monotherapy. Cureus. 2020 Dec 14;12(12):e12086. doi: 10.7759/cureus.12086 | 33489504 | PMC7805509 |
| 1. Qasim Agha O, Kaur S, Vijayavel N. Statin-induced necrotising autoimmune myopathy and autoimmune hepatitis presenting with dysphagia. BMJ Case Rep. 2020 Feb 5;13(2):e232391. doi: 10.1136/bcr-2019-232391 | 32029513 | PMC7021121 |
| 1. Güngör C, Wieshmann UC. Severe statin-induced autoimmune myopathy successfully treated with intravenous immunoglobulin. BMJ Case Rep. 2020 May 21;13(5):e234805. doi: 10.1136/bcr-2020-234805 | 32444443 | PMC7247403 |
| 1. Jasim M, Sapkota H, Timmons M, Manfredonia F, Pohl U, Barkham N. Statin-induced autoimmune necrotizing myositis-A single-center case series highlighting this potentially life-threatening but treatable condition. Clin Case Rep. 2020 Oct 27;8(12):3374-3378. doi: 10.1002/ccr3.3350 | 33363937 | PMC7752384 |
| 1. Lim D, Landon-Cardinal O, Ellezam B, Belisle A, Genois A, Sirois J, Bourré-Tessier J. Statin-associated anti-HMGCR immune-mediated necrotizing myopathy with dermatomyositis-like features: A case report. SAGE Open Med Case Rep. 2020 Dec 29;8:2050313X20984120. doi: 10.1177/2050313X20984120 | 33447390 | PMC7780312 |
| 1. Ghannam M, Manousakis G. Case Report: Immune Mediated Necrotizing Myopathy With IgG Antibodies to 3-Hydroxy-3-Methylglutaryl-Coenzyme a Reductase (HMGCR) May Present With Acute Systolic Heart Failure. Front Neurol. 2020 Nov 25;11:571716. doi: 10.3389/fneur.2020.571716 | 33324322 | PMC7724079 |
| 1. Homas R, Yeoh SA, Berkeley R, Woods A, Stevens M, Marino S, Radunovic A. Initial seronegative immune-mediated necrotising myopathy with subsequent anti-HMGCR antibody development and response to rituximab: case report. BMC Rheumatol. 2020 Jun 30;4:29. doi: 10.1186/s41927-020-00128-5 | 32613157 | PMC7325302 |
| 1. Irvine NJ. Anti-HMGCR Myopathy: A Rare and Serious Side Effect of Statins. J Am Board Fam Med. 2020 Sep-Oct;33(5):785-788. doi: 10.3122/jabfm.2020.05.190450 | 32989074 | NA |
| 1. Mirlesse N, Egervari K, Bornand A, Lecluse J, Lobrinus JA, Scheffler M, Serratrice C, Prendki V, Cuvelier C. Statin-induced autoimmune necrotizing myopathy with pharyngeal muscles involvement. Age Ageing. 2020 Aug 24;49(5):883-884. doi: 10.1093/ageing/afaa038 | 32147707 | NA |
| 1. Close RM, Close LM, Galdun P, Gerstberger S, Rydberg M, Christopher-Stine L. Potential implications of six American Indian patients with myopathy, statin exposure and anti-HMGCR antibodies. Rheumatology (Oxford). 2021 Feb 1;60(2):692-698. doi: 10.1093/rheumatology/keaa337 | 32789452 | NA |
| 1. Stroie OP, Boster J, Surry L. Statin-Induced Immune-Mediated Necrotizing Myopathy: An Increasingly Recognized Inflammatory Myopathy. Cureus. 2020 May 5;12(5):e7963. doi: 10.7759/cureus.7963 | 32523820; | PMC7273436 |
| 1. Soares IFZ, Comprido VF, Hsu BRRHS, Alves de Siqueira Carvalho A. Immune-mediated necrotising myopathy in asymptomatic patients with high creatine kinase. BMJ Case Rep. 2020 Oct 8;13(10):e235457. doi: 10.1136/bcr-2020-235457 | 33033004 | PMC7545499 |
| 1. Revere AS, Appelo B, Bartholomew A, Kuiper B. Weakness Due to Anemia? Go Fish! Melena as a Red Herring in the Diagnosis of Statin-Induced Myopathy. Cureus. 2020 Sep 29;12(9):e10717. doi: 10.7759/cureus.10717 | 33145125 | PMC7598209 |
| 1. Nemati M, Srai M, Rudrangi R. Statin-Induced Autoimmune Myopathy. Cureus. 2021 Feb 26;13(2):e13576. doi: 10.7759/cureus.13576 | 33815984 | PMC8007198 |
| 1. S, Desai K, Rijal S, Zimmerman D. Statin-Induced Autoimmune Necrotizing Myopathy. J Prim Care Community Health. 2021 Jan-Dec;12:21501327211028714. doi: 10.1177/21501327211028714. | 34219515 | PMC8255573 |
| 1. Mak VP, Gravely K, Lim SY. Subcutaneous immunoglobulin therapy in statin-induced necrotizing autoimmune myopathy. Mod Rheumatol Case Rep. 2021 Jan;5(1):58-61. doi: 10.1080/24725625.2020.1782031 | 32967573 | NA |
| 1. Ghaffar MT, Radhakrishna A, Ali I, Whelan B. Statin-induced necrotising autoimmune myopathy: a rare complication of statin therapy. BMJ Case Rep. 2021 Apr 15;14(4):e240865. doi: 10.1136/bcr-2020-240865 | 33858894 | PMC8054048 |
| 1. Ahmad A, Karam I, Baker DL. A Rapidly Debilitating Myopathy: A Rare Case of Statin-Induced Necrotizing Myositis. Cureus. 2021 Jul 10;13(7):e16304. doi: 10.7759/cureus.16304 | 34405065 | PMC8352795 |
| 1. Cha D, Wang F, Mukerji B, Mukerji V. Statin-Induced Necrotizing Autoimmune Myositis: Diagnosis and Management. Cureus. 2021 Mar 9;13(3):e13787. doi: 10.7759/cureus.13787. | 33842161 | PMC8032350 |
| 1. Paul M, Paul P, Dey D, Moazzem SW, Shamrin F. A Case of Statin-Associated Immune-Mediated Necrotizing Myopathy, Successfully Treated With Intravenous Immunoglobulin. Cureus. 2021 Jun 28;13(6):e16001. doi: 10.7759/cureus.16001 | 34336492 | PMC8318619 |
| 1. Ajmal M, Singh A, Kubba S, Hershman M, Acharya T. Statin-Induced Triad of Autoimmune Myocarditis, Myositis, and Transaminitis. Case Rep Cardiol. 2021 Apr 8;2021:6660362. doi: 10.1155/2021/6660362 | 33898067 | PMC8052178 |
| 1. Gupta S, Rakhra A, Thallapally V, Nahas J. Rituximab use for refractory anti-HMGCR immune-mediated necrotizing myopathy: A case report. Intractable Rare Dis Res. 2021 May;10(2):122-125. doi: 10.5582/irdr.2020.03144. | 33996358 | PMC8122313 |
| 1. Torri F, Ali G, Chico L, Siciliano G, Ricci G. Anti-HMGCR antibodies and asymptomatic hyperCKemia. A case report. Acta Myol. 2021 Jun 30;40(2):105-108. doi: 10.36185/2532-1900-050 | 34355128 | PMC8290509 |
| 1. Valdivielso Cortázar E, Delgado Blanco M, Alonso Aguirre P. Dysphagia secondary to autoimmune necrotizing anti-HGMCR myopathy. Rev Esp Enferm Dig. 2021 Oct;113(10):729-730. doi: 10.17235/reed.2020.7324/2020 | 33222486 | NA |
| 1. Sharma A, Musurakis C, Nabil NUN, Poudel B, Trongtorsak A. A Case Series of Statin-Induced Necrotizing Autoimmune Myopathy. Cureus. 2022 Jan 25;14(1):e21613. doi: 10.7759/cureus.21613 | 35233301 | PMC8881230 |
| 1. Ganta N, Alnabwani D, Bommu VJL, Hechter S, Shah V, Cheriyath P. Statin Induced Autoimmune Necrotizing Myopathy (SIANM): An Alarming Adverse Event of a Familiar Medication. Cureus. 2022 Feb 16;14(2):e22273. doi: 10.7759/cureus.22273 | 35350496 | PMC8933139 |
| 1. Smirlis E, Obholz J, Eineichner T, Adio B. A Case of Suspected Statin-Related Delayed Onset Necrotizing Myositis. Cureus. 2022 Mar 6;14(3):e22893. doi: 10.7759/cureus.22893 | 35371858 | PMC8958990 |
| 1. Barrons R, Woods JA, Humphries R. Statin Associated Autoimmune Myonecrosis: Case Report With Delayed Onset and Treatment Challenges. J Pharm Pract. 2022 Feb;35(1):129-134. doi: 10.1177/0897190020958223. | 32924771 | NA |
| 1. Liu L, Tessier S, Ido F, Longo S, Nanda S. Anti-3-Hydroxy-3-Methylglutaryl Coenzyme A Reductase (Anti-HMG CoA) Myopathy With Cardiac Involvement: Presentation, Diagnosis, and Management. Cureus. 2022 Mar 13;14(3):e23125. doi: 10.7759/cureus.23125 | 35425681 | PMC9004697 |
| 1. Alzueta N, Marin M, Castresana M, Gascón A, Pío M, Iguzquiza MJ. Statin-induced autoimmune myopathy: a case report. Eur J Hosp Pharm. 2021 Mar;28(2):115-117. doi: 10.1136/ejhpharm-2019-002102. | 33608442 | PMC7907700 |

References of the articles from which the data of the 67 collected patients have been extrapolated. The unique identifier number used in PubMed for each article (PMID) and PubMed Central identifier (PMCID) have been listed whereas available. N.A: not-available.

**Table 2. Serum complete biochemical exams of the patients presented in our two case reports**

|  | **Patient N.1** | | **Patient N2.** | |
| --- | --- | --- | --- | --- |
| **Biochemical Exams** | **Entry** | **Follow- up** | **Entry** | **Follow-up** |
| Azotemia (mg/dl) (N.V.: 10 – 71) | 40 | 59 | 25 | 25 |
| Creatinine (mg/dl) (N.V.: < 1.2) | 0.62 | 0.57 | 0.45 | 0.57 |
| Sodium (mEq/l) (N.V.: 135 – 147) | 135 | 138 | 136 | 135 |
| Potassium (mEq/l) (N.V.: 3.5 - 5.5) | 4.01 | 4.4 | 4.2 | 4.3 |
| Chlorine (mEq/l) (N.V.: 94 - 110) | 100 | 102 | 99 | 101 |
| Calcium (mg/dl) (N.V.: 8,40 - 10,2) | 10.2 | 9.8 | 8.98 | 9.2 |
| Phosphorus (mg/dl) (N.V.: 2,7 – 4,5) | 4.6 | 3.82 | 3.82 | 4.01 |
| Magnesium (mg/dl) (N.V.: 1,7 – 2,5) | 1.95 | 1.75 | 1.7 | 1.8 |
| Total bilirubin (mg/dl) (N.V.: < 1.2) | 0.75 | N.A. | 17 | 1.19 |
| Gamma GT (U/L) (N.V.: 5-36) | 11 | N.A. | 12 | 10 |
| Alkaline phosphatase (U/L) (N.V.: 35-104) | 43 | N.A. | 52 | 52 |
| Amylase / Lipase (U/L) (N.V.:28-100/13-60) | 41/26 | N.A. | 30/28 | 31/26 |
| **Blood cell count** |  |  |  |  |
| RBC (n/µL) (N.V.:3,8*10^6^-5*10^6^) | 5.44 | 4.30 | 5.11 | 3.98 |
| Hb (g/dL) (N.V.: 12 – 16) | 14 | 11.7 | 15.7 | 13.1 |
| Ht (%) (N.V.: 35 - 48) | 43.9 | 38.4 | 47.5 | 38.2 |
| MCV (fL/g/dL) (N.V.: 80 - 99) | 80.7 | 89.3 | 93 | 96 |
| WBC (n/µL) (N.V.: 4*10^3^-11*10^3^) | 8.63 | 15.22 | 7.41 | 7.19 |
| Neutrophils (%) (N.V.: 40 - 74) | 70.4 | 60.3 | 67.1 | 65.5 |
| Lymphocytes (%) (N.V.: 20 - 48) | 19.6 | 32.7 | 24.4 | 23.9 |
| Monocytes (%) (N.V.: 3 - 11) | 7.6 | 5.3 | 6,1 | 8.8 |
| Eosinophils (%) (N.V.: 0 - 8) | 1.9 | 0.8 | 2 | 1.1 |
| Basophils (%) (N.V.: 0 - 1.5) | 0.5 | 0.9 | 0.4 | 0.6 |
| Platelets (n/µL) (N.V.: 150*10^3^ – 450 *10^3^) | 379 | 356 | 175 | 155 |
| CRP (mg/L) (N.V.: < 5) | 3 | 1.9 | 2.84 | 7.02 |
| INR (N.V.: 0.8-1.2) | 1.02 | N.A. | 0.99 | N.A. |
| APTT (sec.) (N.V.: 22 – 37) | 34 | N.A. | 26 | N.A. |
| Fibrinogen (mg/dl)(N.V.: 150 - 450) | 361 | N.A. | 338 | N.A. |
| Total protein (g/dl) (N.V.: 6,6 – 8,7) | 7.8 | N.A. | 6.47 | N.A. |
| Albumine (g/dl) (N.V.: 3,4 – 4,8) | 4.32 | 3.8 | 4.3 | 3.8 |

Serum biochemical exams at the admission ward and at the last follow-up of both patients (6-month follow-up for patient N.1, 1-year follow-up for patient N.2). RBC: red blood cells, Hb: hemoglobin, Ht: hematocrit, MCV: mean corpuscular volume, WBC: wight blood cells, CRP: c-reactive protein, INR: international normalized ratio, APTT: activated partial thrombosing time.
